# Supplementary material for: Evaluation of Mask Performances in Filtration and Comfort in Fabric Combinations
Source: Nanomaterials (Basel). 2023 Jan 17;13(3):378. doi: 10.3390/nano13030378 (PMC9919832; doi:10.3390/nano13030378)
Supplement: Supplementary file 1 [file nanomaterials-13-00378-s001.zip › nanomaterials-2107714-supplementary.pdf]

Supplementary Information

Evaluation of Mask Performances in Filtration and Comfort in Fabric Combinations

Ji Wang, Renhai Zhao, Yintao Zhao and Xin Ning *

Industrial Research Institute of Nonwovens & Technical Textiles, College of Textiles & Clothing, Shandong Center for Engineered Nonwovens, Qingdao University, Qingdao 266071, China

* Correspondence: xning@qdu.edu.cn; Tel.: +86-532-8595-3572

1. Basic information of filter equipment

The test rig consists essentially of the test channel with the test filter holder, the flow unit, the atomizer aerosol generator and the optical particle measuring device.

Fractional Efficiency of Filters: The fractional separation efficiency and particle size distributions can be determined separately using a laser aerosol particle size spectrometer LAP 322 (optical particle counter/sizer) in a particle size range of 0.2 to 40 microns.

Volume flow rate 2~40 m^3/h

Filter cross section 176 cm^2 (150 mm)

Face velocity 0.05~0.6 m/s

Differential pressure 0~2000 Pa

Droplet aerosols: DEHS and salt solutions with Atomizer Aerosol Generator ATM 220

Aerosol Spectrometer: LAP 322, 0.2~40 μm <104 particles/cm^3

Selected phrases: dust penetration through the filter medium; in accordance with international standards (the VDI 3926 Part 1, Version 2); air filters for air conditioning systems according to EN 779; air filters for motor vehicles according to DIN 71460-1 / ISO/TS 11155-1; the test rig; accurate, repeatable and traceable testing; Automated Filter Media Test Rig AFC 131; ambient air inlet; test channel; Pressure drop across the filter is measured at 4 points over the filter holder. The flow rate unit uses a mass flow controller and is designed to keep the flow rate constant within 2% of the set value throughout the complete test procedure.

2. Formula for calculating moisture permeability

WVT = (△m-△m ')/A*t

WVT -- Moisture permeability, in grams per square meter hour [g/(m2*h)]; △m -- the difference between two weights of the same test assembly, expressed in grams (g); △m'-- the difference between two weights of the same test assembly in the blank test, in grams (g). In this study, △m' =0; A -- Effective test area (in this part, the device is 0.00283m^2), the unit is square meters (m^2); t -- Test time, expressed in hours (h).

The relation between surface hairiness and fabric filtration performance.

3. Characteristics and structural properties of the fabric

Table S1. Characteristics and structural properties of the fabric.

Sample	Density (piece/10 cm)		Diameter of yarn (μm)		Fabric tightness (%)		Tensile stress（MPa）		Tensile strain（%）	
	Warp	Weft	Warp	Weft	Warp	Weft	Warp	Weft	Warp	Weft
Cotton3	190	250	297.4	353.8	56.5	88.4	4.2	9.5	26	25.5
Wool1	250	200	333.1	290	83.3	58	4.9	5.5	31.2	29

Polyester2	200	300	204.9	165.6	41	49.7	7.9	16.4	42.7	55.3
Silk1	220	200	245.6	141.7	54	28.3	16.8	26.8	33.8	13.2
Blend1	300	300	193.8	170.7	58.1	51.2	30	51.2	18.1	48.2
Blend2	200	120	371	154.8	74.2	18.6	6.5	6	30.6	19.2

4. The dynamic water contact angle of Cotton3 and Silk 1

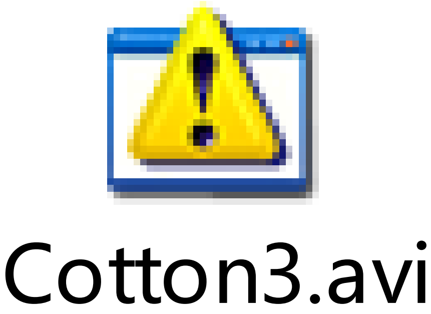

Video S1. The dynamic water contact angle of Cotton3 fabric.

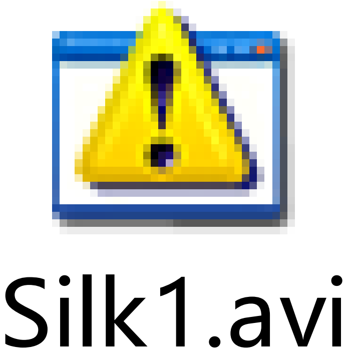

Video S2. The dynamic water contact angle of Silk 1 fabric.

5. The friction process of silk.

In the same temperature and humidity conditions (temperature 24℃, humidity 40%RH), wear latex gloves friction sample for 30 s, friction immediately after the sample into the filter material test bench, test and record the filtration performance before and after treatment. Considering that NaCl is an ionic compound, ions in aerosols will neutralize charged particles on the sample surface, we used DEHS (Polar non-ionic compound) aerosols for testing.

6. The electrospinning process of PLA fibers.

The raw material of the selected non-woven fabric was left-handed PLA with a concentration of 8%. The spinning solution was equipped with methylene chloride (DCM) and dimethylformamide (DMF) as solvents. The solution was stirred at room temperature for eight hours before use. Spinning conditions: Under the condition of temperature between 30℃ and 39℃, the stirred solution was put into a 10 ml syringe, a needle with an inner diameter of 0.42 mm was used, the receiving distance between the syringe and the

drum was 17 cm, the injection flow rate was set as 1 mL/h, and the rotation speed of the drum was adjusted to 350 rpm/min, finally add a positive voltage at the needle, and apply a voltage of 20 kV.

Experimental conditions: RH 30%~40%, temperature 20±2 ℃, power supply voltage is 220V/50HZ, discharge voltage is 10 kV, the discharge distance between needle electrode and style is 20 mm, test probe and test spacing between 15 mm, sample size is 60 mm*80 mm, style number is 3 pieces, The samples were pre-dried for 30 min at 50 ℃ and tested for 5 h after debugging and balancing under atmospheric conditions.

7. The basic structure and filtration parameters of different non-woven fabrics

Table S2. The basic structure and filtration parameters of different non-woven fabrics.

Sample	SEM	Thick-ness (mm)	Fiber diame-ter (um)	Average aperture (um)	Gram weight (g/m²)	Filtration efficiency		Pressure drop (Pa)
						0.3μm	2.5μm	
PLA spun-bonded 1	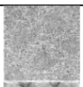	0.25	15.52	66.39	40	42	81	3
PLA spun-bonded 2	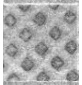	0.39	13	37.63	80	56	90	9
PP spun-bonded 1	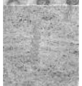	0.26	13.95	60.51	50	7	37	7
PP spun-bonded 2	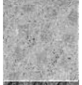	0.21	13.46	38.86	50	6	40	10
PP three layer bonding 1	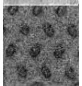	0.28	15.06/	10.44	47	31	87	26
PP three layer bonding 2	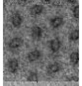	0.33	15.13/	12.09	47	42	90	23
Split fiber 1 （PET/PA=7/3）	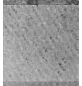	0.24	7.69	31.25	68	53	78	20
Split fiber 2 （PET/PA=5/5）	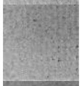	0.31	7.22	20.98	80	58	88	40
PLA melt blown	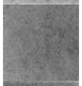	0.15	1.92	14.87	30	90	100	18
PLA electrospinning	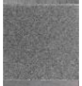	0.01	0.47	11.03	4	94	100	8
PP melt blown	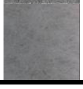	0.17	2.42	16.59	21	93	99	19

8. The effect of surface hairiness on the mask material filtration properties

In order to further explore the influence of fabric surface hairiness on fabric filtration performance, the surface hairiness of Blend 2 fabric was trimmed 5 times and 10 times via the hair removing machine. And the filtration performance of the treated sample was compared with the original sample. The results were shown in Figure S1. Figure S1 (a, b, c) respectively show the thickness and pressure drop of the of original, trimmed 5 times and trimmed 10 times fabric respectively. It can be seen that the fabric becomes thinner with the increase of the number of removing, but the main tissue of the fabric will not be damaged, so the pressure drop basically does not change. As can be seen from Figure S1 (d) and (e), the filter efficiency of the three fabrics decreases with the increase of the trimming times, whether as a whole or as a single particle size. Because there are more feathers on the surface of the original sample, which can close the pores on the surface. And the aerosols have more opportunity to collide with the single fiber and be caught. When the

particles pass through the fiber layer with the air flow, they repeatedly go through fierce turns. Due to their inertia, the moving particles have no time to follow the changes of the streamline and bypass the fiber, so they are caught by the fiber. Figure S1f shows the filtration efficiency and quality factor at 0.3 um and 2.5 um respectively. Because the filtration efficiency and pressure drop of mixture 2 are slightly reduced, the quality factor is slightly reduced.

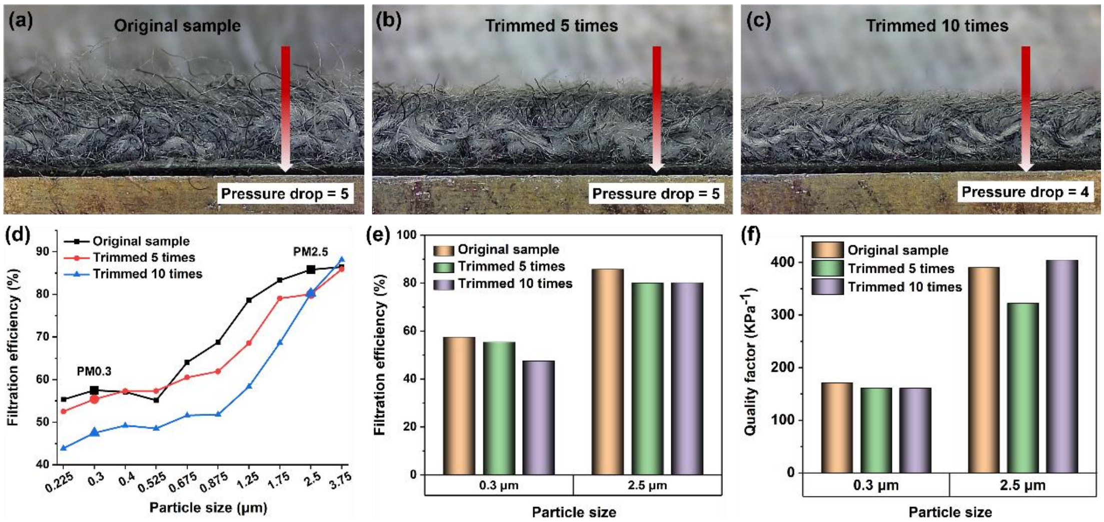

Figure S1. (a-c) The microphotograph and (d-f) the filtration performance graph of the original, trimmed 5 times and trimmed 10 times fabric.

9. The effect of washing on the mask material filtration properties

Cotton 3 and wool 1 are put through a washing and drying cycle in a household washing machine with the same parameters as for washing clothes. The figure shows the comparison of filtration performance of cotton and wool fabric before and after washing. The quality factor (Figure S2c) decreases slightly at particle size of 0.3 μm and increases slightly at particle size 2.5 μm. The average pore diameter of Cotton 3 is 47.3 μm before washing and 58.5 μm after washing. The pore size of wool 1 was 33.7 μm before washing and 43.1 μm after washing (Figure S2d). Therefore, the filtration efficiency and pressure drop were decreased (Figure S2a, b). When the filter efficiency is 0.3 μm, it can be seen that washing has a small negative effect on the fabric blocking, which may be due to the dissipation of fabric surface charge, the attraction of small particle size particles is weakened, but a little effect on the filtration of 2.5 μm particles.

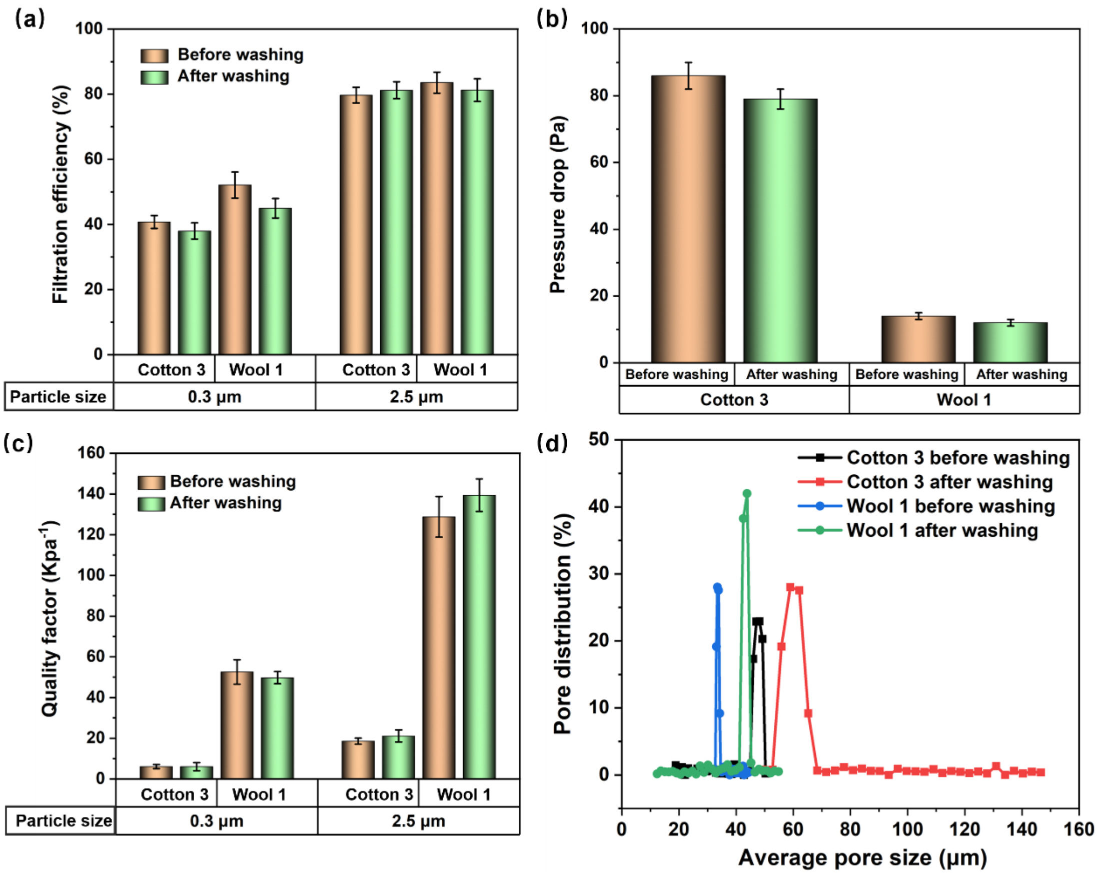

Figure S2. (a) The filtration efficiency, (b) pressure drop, (c) quality factor and (d) pore size graph of Cotton 3 and Wool 1 fabrics before and after washing.
